# Traveling EEG slow oscillation along the dorsal attention network initiates spontaneous perceptual switching

**DOI:** 10.1007/s11571-012-9196-y

**Published:** 2012-03-21

**Authors:** Takashi J. Ozaki, Naoyuki Sato, Keiichi Kitajo, Yoshiaki Someya, Kimitaka Anami, Hiroaki Mizuhara, Seiji Ogawa, Yoko Yamaguchi

**Affiliations:** 1 Laboratory for Dynamics of Emergent Intelligence, RIKEN Brain Science Institute, Wako, Saitama Japan; 2 Department of Life Sciences, Graduate School of Arts and Sciences, University of Tokyo, Building No. 2, Room 105A, 3-8-1 Komaba, Meguro-ku, Tokyo, 153-8902 Japan; 3 Department of Complex Systems, School of Systems Information Science, Future University Hakodate, Hakodate, Hokkaido Japan; 4 Rhythm-based Brain Computation Unit, BSI-Toyota Collaboration Center, RIKEN Brain Science Institute, Wako, Saitama Japan; 5 Laboratory for Cognitive Brain Mapping, RIKEN Brain Science Institute, Wako, Saitama Japan; 6 PRESTO, Japan Science and Technology Agency (JST), Kawaguchi, Saitama Japan; 7 Ogawa Laboratories for Brain Function Research, Hamano Life Science Research Foundation, Shinjuku-ku, Tokyo, Japan; 8 Global COE Program Center for Advanced Research on Logic and Science, Keio University, Minato-ku, Tokyo, Japan; 9 Ohmiya Musashino Clinic, Saitama, Saitama Japan; 10 Department of Intelligence Science and Technology, Graduate School of Informatics, Kyoto University, Kyoto, Kyoto Japan; 11 Kansei Fukushi Research Center, Tohoku Fukushi University, Sendai, Miyagi Japan

**Keywords:** EEG, fMRI, Simultaneous EEG–fMRI, Dorsal attention network, Multistable perception, Slow oscillation, Intrinsic neural dynamics

## Abstract

An ambiguous figure such as the Necker cube causes spontaneous perceptual switching (SPS). The mechanism of SPS in multistable perception has not yet been determined. Although early psychological studies suggested that SPS may be caused by fatigue or satiation of orientation, the neural mechanism of SPS is still unknown. Functional magnetic resonance imaging (fMRI) has shown that the dorsal attention network (DAN), which mainly controls voluntary attention, is involved in bistable perception of the Necker cube. To determine whether neural dynamics along the DAN cause SPS, we performed simultaneous electroencephalography (EEG) and fMRI during an SPS task with the Necker cube, with every SPS reported by pressing a button. This EEG–fMRI integrated analysis showed that (a) 3–4 Hz spectral EEG power modulation at fronto-central, parietal, and centro-parietal electrode sites sequentially appeared from 750 to 350 ms prior to the button press; and (b) activations correlating with the EEG modulation traveled along the DAN from the frontal to the parietal regions. These findings suggest that slow oscillation initiates SPS through global dynamics along the attentional system such as the DAN.

## Introduction

Seeing is a process of uniquely choosing a single percept from among many possibilities. When seeing a single ambiguous figure that allows multiple percepts, however, an observer’s representation cannot always be embodied uniquely; our percept easily changes from one percept to another without any physical change in the figure. The Necker cube, Rubin’s vase (Leopold and Logothetis 1999), and the dynamic dot quartet (Ramachandran and Anstis 1983) are examples that induce these phenomena. A transient process of this phenomenon is spontaneous perceptual switching (SPS), in which observers switch spontaneously from one percept to another, indicates that complex neural mechanisms underlie multistable perception.

Functional magnetic resonance imaging (fMRI) has shown that the dorsal frontoparietal regions are involved in SPS induced by the Necker cube (Inui et al. 2000; Shen et al. 2009). These dorsal frontoparietal regions, also called the “dorsal attention network” (DAN), are related to voluntary attentional control (Corbetta and Shulman 2002; Corbetta et al. 2008). Moreover, experiments have shown the involvement of the DAN in both Necker-cube induced SPS and voluntary attentional control (Slotnick and Yantis 2005), suggesting that these processes are closely related and involve common neural substrates. This hypothesis is consistent with the results of a psychophysical study, which found that SPS induced by the Necker cube could be modulated by voluntary attentional control, whereas binocular rivalry could not (Meng and Tong 2004).

Furthermore, recent effective connectivity fMRI studies with optimized experimental (longer duration of events) and imaging (longer repetition time of fMRI scanning) parameters for effective connectivity analysis, such as Granger causality analysis, indicated that voluntary attentional control may be related to top-down connectivity of neural processing along the DAN (Bressler et al. 2008; Ozaki and Ogawa 2009; Ozaki 2011).

These findings lead to another hypothesis that similar top-down connectivity of neural processing along the DAN may also occur in case of SPS induced by the Necker cube, because the DAN may be a common neural substrate of both voluntary attentional control and SPS induced by the Necker cube, as suggested by previous behavioral and functional neuroimaging studies.

On the other hand, the mechanism by which neural processes initiate SPS still remains unclear. Especially, because of the low temporal resolution of fMRI and the excessively rapid changing behavior of Necker cube induced SPS, fMRI alone could not determine this mechanism even using recently developed effective connectivity analysis techniques.

In contrast, electroencephalography (EEG) has revealed neural correlates of Necker cube induced SPS cube in a fine time scale (Kornmeier and Bach 2004, 2005; Britz et al. 2009). Moreover, some oscillatory EEG activities, especially delta-band (around at 4 Hz) activities, have been shown to correlate with SPS (Nakatani and van Leeuwen 2005, 2006; Shimaoka et al. 2010; Nakatani et al. 2011). Delta-band oscillatory EEG activities have been thought to be mediators of long-range cortical networks such as the DAN (Isoglu-Alkac et al. 1998, 2000; Mathes et al. 2006). These findings suggest that some neural dynamics may be self-organized by slow oscillatory neural activities in the human brain, in agreement with theoretical predictions (Shimizu and Yamaguchi 1987; Yamaguchi and Shimizu 1994). Furthermore, a previous EEG study even indicated that the frontal-to-occipital synchrony or connectivity may be important for SPS induced by the Necker cube (Shimaoka et al. 2010). On a larger level, the fronto-parietal delta-band oscillatory EEG coherence was suggested as an important component for a general attention-demanding cognition (Guntekin and Basar 2010). This line of evidence appears to support our current hypothesis.

Recent advances in imaging techniques have enabled the simultaneous recording of EEG data on a fine time scale during fMRI scanning, allowing us to determine the dynamics of neural activity on a fine time scale (Mizuhara et al. 2004, 2005; Mizuhara and Yamaguchi 2007; Laufs et al. 2008). These findings indicate that simultaneous EEG–fMRI technology may be a promising method for testing our hypothesis.

To determine whether rapidly changing neural dynamics along the DAN from anterior (frontal) to posterior (parietal) regions occur prior to Necker cube induced SPS, we performed a simultaneous EEG–fMRI experiment with EEG–fMRI integrated analysis and supplementary EEG signal source estimates on subjects performing an SPS task with the Necker cube.

## Methods

### Participants

Twenty right-handed healthy volunteers (21–44 years old, 4 females, normal or corrected-to-normal visual acuity) participated in the experiment and each participant gave written informed consent for participation. The experiment and its protocol were approved by the Institutional Review Boards of RIKEN and Ogawa Laboratories for Brain Function Research, respectively.

### Stimuli and experimental procedure

**Fig. 1:**
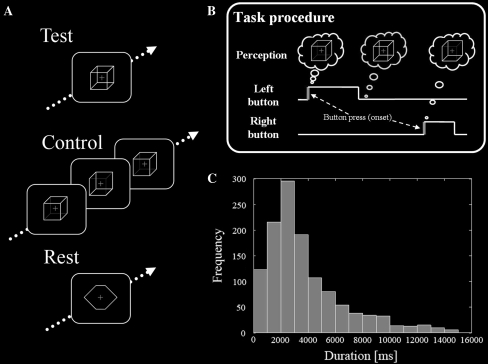
Stimuli, task procedure, and behavioral results. **a** Schematic depictions of visual stimuli. (*Top*) test condition, in which the Necker cube appeared continuously, (*middle*) control condition, in which a pair of opaque cube stimuli appeared alternatively, with *hidden lines* represented as *dimmed lines*, (*bottom*) rest condition, in which a hexagon with the same outline as the Necker or opaque cubes appeared continuously. **b** A task procedure. Participants were instructed to hold one of the two buttons, corresponding to one of the two viewpoints of the Necker or opaque cubes, until an alternative percept arose. **c** Histogram of duration of button pressing in the test condition, showing a gamma distribution (see text)

Each experimental session consisted of three blocks, each with its own condition. In the test condition, a Necker cube (size: 4.5° × 4.5°) was presented continuously with a fixation cross (Fig. 1a). In the control condition, a pair of cube stimuli (4.5° × 4.5°) with different viewpoints was presented alternately. In both the test and control conditions, participants were instructed to gaze at the fixation cross and respond by holding down one of two buttons until the next percept arose, thus allowing the participant to indicate the viewpoint from which he/she perceived the cube (left button, upper viewpoint; right button, lower viewpoint; Fig. 1b). In the rest condition, participants passively viewed a hexagon with the same outline as the Necker cube and normal cube stimuli, but did not make any responses. Each block of condition lasted 3 min 6 s; therefore, each session lasted 9 min 18 s. Each participant was involved in three sessions per experiment.

A pilot study indicated that seeing a pair of cube stimuli was influenced by the experience of seeing a Necker cube; therefore, in these experiments, blocks of the test condition always preceded those of the control condition. To counterbalance the effect of a fixed order of blocks and to avoid overestimation of activation by fMRI, a mask procedure of the activation map was performed (see EEG–fMRI integrated analysis).

### Simultaneous EEG data recording with fMRI and preprocessing

During fMRI scanning, scalp EEG was simultaneously recorded using a Brain Cap MR 64 Ag/AgCL electrode cap with two Brain Amp MR (Brain Products, Gilching, Germany) 32 channel amplifiers. Impedances were kept below 5 kΩ at the ground and reference electrodes and below 20 kΩ at other electrodes. EEG data were digitized at a sampling rate of 5,000 Hz and filtered with 1.0 Hz highpass and 250 Hz lowpass filters. Data obtained from the third session of all participants were excluded because of low data quality. Data from the second session of two participants were excluded because of excessive body movements.

Prior to data analysis, the recorded EEG data were preprocessed by Brain Vision Analyzer software (Brain Products) to reduce artifacts derived from the simultaneous EEG–fMRI environment. First, MRI scanning artifacts were reduced by the averaged artifact subtraction (AAS) method (Allen et al. 2000), with the EEG amplifiers and MRI scanner synchronization technique (Mandelkow et al. 2006). Second, ballistocardiographic artifacts were reduced by the AAS method (Allen et al. 1998). Finally, any remaining slice scan timing artifacts and unidentified machinery artifacts were reduced by the phase-shift free band-cutoff filter. Cutoff frequencies were 16.0 and 19.33 Hz, including harmonics of 19.33 Hz in the higher frequency bands. 16.0 Hz was a frequency of a persistent artifact occurring even in case of simultaneous EEG–fMRI recording with a saline phantom and it was suspected as the scanner-specified machinery artifact.

### EEG data analysis

Preprocessed EEG data were analyzed by Brain Vision Analyzer software and Matlab software package (Mathworks, Natick, MA). The data were downsampled to a 250 Hz sampling rate and filtered with a 1–40 Hz bandpass filter. The reference used for the EEG data was changed to the average of all electrodes after filtering because ear reference electrodes showed low signal-to-noise ratios, due to ear mufflers that protected participants’ ears from fMRI scanning noises. This process was followed by infomax independent component analysis (ICA) to the filtered data to segregate noises and artifacts from true EEG signals, because any remaining noises or artifacts would cause severe artifacts of EEG-derived regressors for fMRI data analysis (Delorme and Makeig 2004). By visual inspection, we excluded independent components (ICs) with (a) waveforms that correlated with the electrooculograms, (b) abnormally widely distributed frequency spectra, (c) spike-shaped waveforms causing broadly-distributed gamma-band artifacts (Yuval-Greenberg et al. 2008), (d) a reconstructed EEG topography showing prominent left–right symmetry with greater amplitude (Britz et al. 2010). Consequently, 28 ± 3 independent components were excluded from the EEG data of each participant. We next computed wavelet transforms (complex Morlet’s wavelet with a constant ratio of f/σ_f_ = 7) of the EEG data to obtain time courses of spectral power variations in the time–frequency domain. Finally, to evaluate event-related modulation, the spectral power variations of the time–frequency domain were averaged and time-locked to the button holding onset latency.

Wavelet-transformed EEG data were statistically analyzed based on the random effects model. All EEG data were normalized relative to the EEG data in the rest condition. For first level analysis, a two-sample, two-tailed t-test was performed on each participant to quantify the modulation in the test condition compared with the control condition. For second level analysis, a one-sample, two-tailed t-test was performed on t-values from all participants using a random effects model, with the significance threshold set at an uncorrected *p* < 0.05.

### EEG signal source estimates

In order to obtain a rough picture of neural dynamics from the EEG data, the standardized low-resolution brain electromagnetic tomography (sLORETA) inverse solution was performed with the distributed software package (Pascual-Marqui 2002), onto the EEG data simultaneously recorded during the fMRI scanning.

For the sLORETA inverse solution, first statistically significant oscillatory EEG component in time–frequency domain was extracted as a difference of time course of oscillatory EEG power between the test and control condition. Second, the extracted time course was averaged across all time points in order to stabilize source estimates in terms of time. Finally, the inverse solution was performed with a solution space defined by the relative head-surface-based approximation of the 10/10 electrodes system coordinates (Jurcak et al. 2007), onto the digitized probability brain atlas of the Brain Imaging Center at Montreal Neurological Institute (Collins et al. 1994).

### fMRI data acquisition and preprocessing

A Magnetom Allegra 3.0 T head-dedicated MRI scanner (Siemens, Erlangen, Germany) was used to acquire fMRI data, based on the blood oxygenation level dependent (BOLD) effect (Ogawa et al. 1992). For each participant, functional images were scanned using a T2*-weighted single-shot echo planar imaging (EPI) sequence. Scanning parameters were: TR = 1,500 ms, TE = 22 ms, flip angle = 90°, field of view = 230 × 230 mm^2^, imaging matrix = 64 × 64, and in-plane resolution = 3.0 mm. These images consisted of 29 contiguous transverse slices covering the cerebral cortex (not including the cerebellum) with no gaps, with 375 whole-brain volumes acquired during each session. T1-weighted high-resolution anatomical images were scanned as sagittal slices with the magnetization-prepared rapid-acquisition gradient echo (MPRAGE) sequence (TR = 2,500 ms, TE = 4.38 ms, voxel size = 1.3 × 1.0 × 1.0 mm).

Acquired functional images were preprocessed by BrainVoyager QX software (Brain Innovation, Maastricht, Netherlands). The first three volumes of each scan were discarded to allow the magnet to stabilize. The remaining 372 volumes were filtered at a three cycle per session temporal frequency to remove any linear trend. The volumes were realigned to the first image, corrected for head motion and differences in slice scan timing, smoothed using a 7.0 mm full-width half maximum Gaussian spatial filter, and normalized to Talairach stereotaxic space (Talairach and Tournoux 1988).

### EEG–fMRI integrated analysis

To determine the correlation between electrophysiological oscillatory activities and hemodynamic neural activations as neural substrates of SPS, two general linear models (GLM) were computed to obtain activation maps. One model was based on the block effects defined by a box-car function of three experimental blocks (test, control and rest, ‘model 1’). The other model was based on variations of EEG spectral power at a specified frequency, the onsets of participants’ responses, and block effects. In the first model, each regressor was computed by convolution of each box-car function with the canonical hemodynamic response function (HRF) in the blocked-design manner as a “block-effect” regressor (‘model 2’). The latter model utilized three steps to compute regressors. First, an EEG based regressor was obtained by convolution of the time course of the normalized EEG spectral power variation at a specified frequency and electrode with the canonical HRF. Second, a response-onset based regressor was computed by convolution of the time course of the button onset timings with the canonical HRF. Finally, the arithmetic sum of the EEG based regressor, the response-onset based regressor, and the block-effect regressor was computed as an “EEG-motor-block” combined effects regressor (Mizuhara et al. 2004, 2005; Mizuhara and Yamaguchi 2007). The response-onset based regressor was included in the EEG-motor-block effect regressor since explicit motor responses are not included in the EEG data. Thus, information about motor responses should be obtained from behavioral data, independent of EEG data.

After two models were specified, one activation map was computed by model 1 as a block-effect map, and the other activation map was computed by model 2 as an EEG-motor-block effect map. For both maps, a linear contrast as [test > control] was used to compute voxel-wise statistics (random effects model, uncorrected *p* < 0.005, volume threshold as 40 mm^3^). Finally, to counterbalance the effect of the fixed order of the experimental blocks and to avoid overestimation of activation, the model 2-derived map was masked by the model 1-derived map (Mizuhara et al. 2004, 2005; Mizuhara and Yamaguchi 2007).

## Results

### Behavior

In the experiment, twenty participants performed an SPS task with the Necker cube during fMRI scanning. Data from four participants were discarded because of excessive artifacts in EEG and/or fMRI data caused by body movements or problems with machinery. Thus, data from 16 participants were analyzed and evaluated.

A histogram of the perceptual duration in the test condition showed a trend of gamma distribution; the histogram could be fitted by a gamma distribution Ga(k, θ) where k = 1.62 and θ = 2,494.9 (Fig. 1c). The correlation of determination of the fitted gamma distribution (R^2^) was 0.908, consistent with the results of a previous psychological study (Murata et al. 2003). In contrast, R^2^ between the distribution of the response duration and that of the stimulus duration in the control condition was 0.796. This result indicates that the participants adequately performed the task in the control condition and successfully followed viewpoint changes of the cube stimuli. The mode value of the response time to changes in the control stimuli was 357 ms. This latency can be regarded as a stimulus onset latency of SPS induced by the Necker cube.

### EEG oscillatory dynamics prior to SPS

**Fig. 2:**
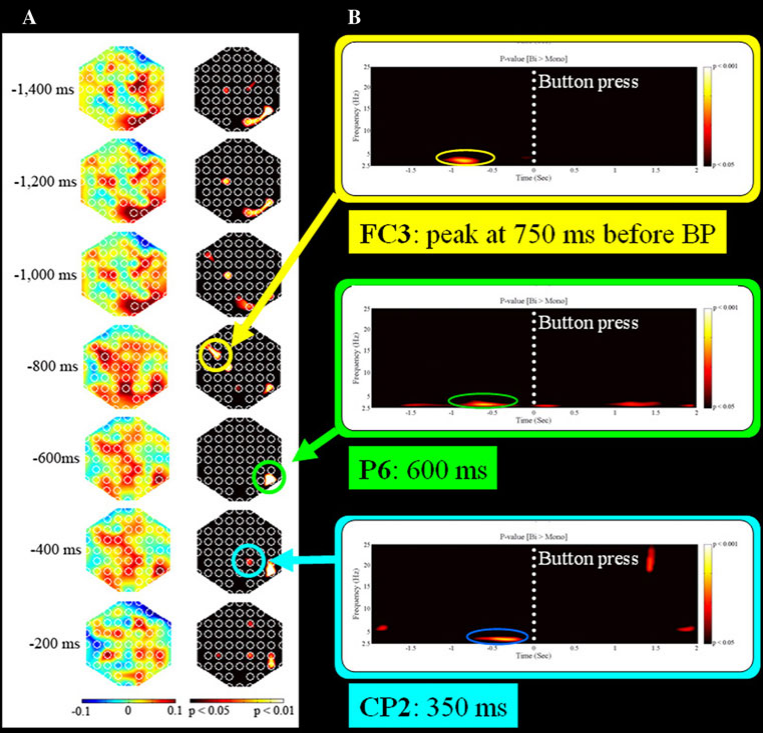
EEG data recorded simultaneously during fMRI scans. **a** Time course of the EEG topographic map in the time–frequency domain from 1,400 to 200 ms before button pressing. (*Left column*) difference in normalized EEG power between the test and control conditions. (*Right column*) T values calculated by an unpaired t test comparing the normalized EEG power of the test and control conditions. **b** Time–frequency domain plots of T values at the FC3 (*top*), P6 (*middle*), and CP2 (*bottom*) electrodes. Peak T values were observed at 750, 600, and 350 ms, respectively, before the button was pressed

To determine the detailed time course of neural dynamics related to SPS during multistable perception, we performed a time–frequency domain analysis of spectral power in the simultaneously-recorded EEG and fMRI data in an event-related manner. The time–frequency domain plots time-locked by the button-press response showed a significantly greater modulation at lower frequency bands associated with SPS in the test than in the control condition. Topographic maps of the EEG spectral power at 3.0 Hz from 1,400 to 200 ms before the button was pressed showed significant modulations at the left frontal, right parietal and centro-parietal electrodes in the test compared with the control condition (Fig. 2a).

We chose three electrodes showing prominent modulation at approximately 3–4 Hz narrow frequency band: FC3, P6, and CP2. Time–frequency domain plots of the EEG data recorded at these three electrodes demonstrated significant modulations at 2.5–4.4 Hz with a peak at 750 ms before the button press for FC3 electrode (abbreviated as “early FC3 component”: Fig. 2b, top), at 2.6–3.4 Hz with a peak at 600 ms before the button press for P6 electrode (“middle P6 component”: Fig. 2b, middle), and at 2.8–4.0 Hz with a peak at 350 ms before the button press for CP2 electrode (“late CP2 component”: Fig. 2b, bottom). These oscillatory EEG components appeared to correlate with SPS.

From the behavioral data in the control condition, 357 ms before the button press was estimated as an “assumed onset of the percept of a cube” in both the test and control condition. The relationship of the latencies of the three oscillatory EEG components implied that the early FC3 component and the middle P6 component arose prior to the assumed percept of the Necker cube, and that only the late CP6 component occurred almost at the same time with the percept.

### Low spatial resolution neural dynamics from the anterior to posterior cortical regions estimated from the oscillatory EEG data

Prior to EEG–fMRI integrated analysis with high temporal resolution, time-varying electromagnetic source estimates were performed by using the standardized low-resolution electromagnetic tomography (sLORETA) method. For the source estimates, each oscillatory EEG component was extracted as follows: 1,100–750 ms before the button press at 2.5–4.4 Hz for the early FC3 component, 800–400 ms at 2.6–3.4 Hz for the middle P6 component, and 750–200 ms at 2.8–4.0 Hz for the late CP2 component based on the *p*-value plot in time–frequency domain (Fig. 2).

**Fig. 3:**
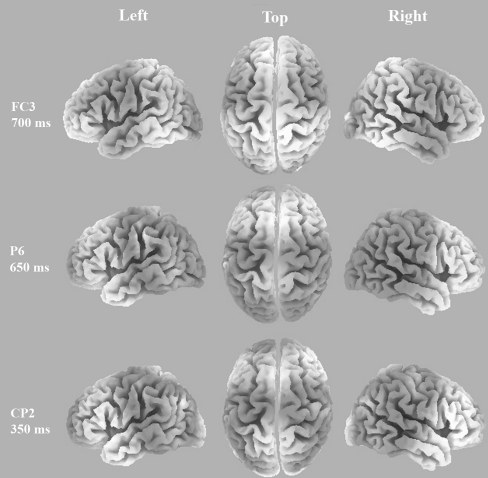
sLORETA solution maps for each of three oscillatory EEG components projected onto a reconstructed and 3D-rendered atlas cerebral volume (Collins et al. 1994)

Figure 3 showed results of the sLORETA source estimates for the three oscillatory EEG components. Despite its low spatial resolution, the results indicated neural dynamics before and/or at SPS. For the early FC3 component, the sLORETA solution showed broader bilateral frontal distribution covering and focal bilateral occipital distribution. For the middle P6 component, the source estimates solution showed broader bilateral post-central-parietal distribution. Finally, for the late CP2 component, the source estimate solution indicated broader bilateral parietal distribution.

In total, the three sLORETA solutions indicated a qualitative trend in which neural dynamics before and/or at SPS traveled from the anterior to posterior cortical regions, while the solution for the early FC3 component included narrow but rather intensive occipital distribution.

### Cortical activations correlating with EEG oscillatory dynamics prior to SPS revealed by EEG–fMRI integrated analysis

The result of the sLORETA solutions indicated a trend of neural dynamics traveling from the anterior to posterior cortical regions. However, it cannot precisely localize details of neural activity yielding such neural dynamics. In the current study, we applied EEG–fMRI integrated analysis to the fMRI data in order to localize the neural activity underlying the neural dynamics indicated by the sLORETA solutions.

**Fig. 4:**
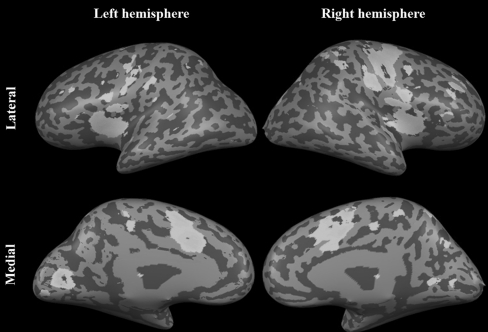
An activation map of the effect of experimental blocks with a linear contrast as [test block > control block] (random effects model, uncorrected *p* < 0.005, volume threshold as 40 mm^3^). This activation map was used to compensate the effect of the fixed block order. See Table 1 for details of the activated clusters

To obtain fMRI activation maps with EEG–fMRI integrated analysis, we first utilized a conventional blocked design analysis. Consistent with previous fMRI results, our activation map with a linear contrast as [test > control] showed considerable activation at the premotor, inferior frontal, medial frontal, dorsal parietal, and occipital regions of both hemispheres (Inui et al. 2000). This activation map was used to compensate for the effect of experimental blocks, since the block order was fixed, not randomized (see Materials and Methods). Figure 4 showed the activation map of the effect of experimental blocks. A summary of activated clusters is shown in Table 1.

**Table 1:** Talairach coordinates of brain clusters

Cluster name	Talairach coordinate (mm)			Volume (mm^3^)
	x	y	z	
R MFG	36	41	32	473
R aIC	36	12	8	1,089
R mFC	9	−1	50	1,037
R PrCeS	27	−11	52	1,210
R PoCeG	57	−16	33	812
R PCC	14	−32	47	528
R IPS	29	−48	52	563
R Cal	27	−48	5	159
L MFG	−32	31	32	591
L aFO	−33	15	8	904
L mFC	−4	0	50	855
L pFO	−41	−4	10	895
L PrCeG	−41	−8	43	513
L PCC	−8	−32	47	520
L Cal	−7	−75	6	664
L Extr	−22	−76	−7	58
ACC	2	14	33	1,047

*R* right, *L* left, *MFG* middle frontal gyrus, *aIC* anterior insular cortex, *mFC* medial frontal cortex, *PrCeS* precentral sulcus, *PoCeG* postcentral gyrus, *PCC* posterior cingulated cortex, *IPS* intraparietal sulcus, *Cal* calcarine sulcus, *aFO* anterior frontal operculum, *pFO* posterior frontal operculum, *PrCeG* precentral gyrus, *Extr* extrastriate cortex, *ACC* anterior cingulated cortex

**Fig. 5:**
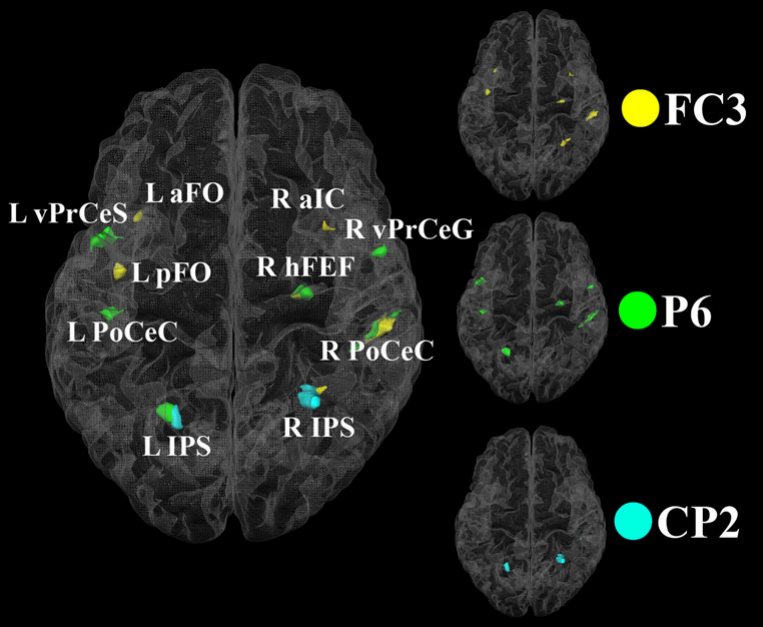
Activation maps provided by the EEG–fMRI integrated analysis. Three overlaid activation maps provided by the EEG–fMRI integrated analysis using EEG data recorded at the FC3 (*yellow*), P6 (*green*) and CP2 (*blue*) electrodes. See the caption of Table 2 for abbreviations of the activated clusters. (Color figure online)

We subsequently performed an EEG–fMRI integrated analysis based on the time variation of the spectral power of the EEG data recorded at the FC3, P6 and CP2 electrodes, counterbalancing the effects of experimental blocks and motor responses in order to avoid overestimation of activation (see Materials and Methods). The activation maps with a linear contrast as [test > control] showed focused activations at each of the three electrodes (see the caption of Table 2 for definitions of the activation sites): the FC3 group activation sites at 2.5–4.4 Hz with a peak at 750 ms included the R hFEF, R IPS, R PoCeC, R aIC, L aFO and L pFO sites; the P6 group activation sites at 2.6–3.4 Hz with a peak at 600 ms included the R hFEF, L IPS, L PoCeC, R PoCeC, R vPrCeG and L vPrCeS sites; and the CP2 group activation sites at 2.8–4.0 Hz with a peak at 350 ms included the R IPS and L IPS sites. All activation sites are shown in Fig. 5, and their details are described in Table 2.

**Table 2:** Talairach coordinates of clusters corresponding to the FC3, P6, and CP2 electrodes

ROI name	Talairach coordinate (mm)			Volume (mm^3^)
	x	y	z	
FC3-group				
R aIC	34	12	9	47
R hFEF	26	−12	53	119
R PoCeC	53	−24	33	233
R IPS	28	−49	52	163
L aFO	−34	16	8	52
L pFO	−41	−4	10	88
P6-group				
R vPrCeG	53	3	27	107
R hFEF	25	−12	53	209
R PoCeC-2	54	−23	32	209
R PoCeC-1	48	−28	40	104
L vPrCeS	−47	8	14	378
L PoCeC	−43	−19	29	97
L IPS	−23	−55	48	192
CP2-group				
R IPS	28	−49	52	343
L IPS	−21	−56	49	151

*R* right, *L* left, *aIC* anterior insular cortex, *hFEF* human homologue of the frontal eye field (at the conjunction of the precentral sulcus and superior frontal sulcus), *PoCeC* postcentral cortex, *IPS* intraparietal sulcus, *aFO* anterior frontal operculum, *pFO* posterior frontal operculum, *vPrCeG* ventral precentral gyrus, *vPrCeS* ventral precentral sulcus

## Discussion

**Fig. 6:**
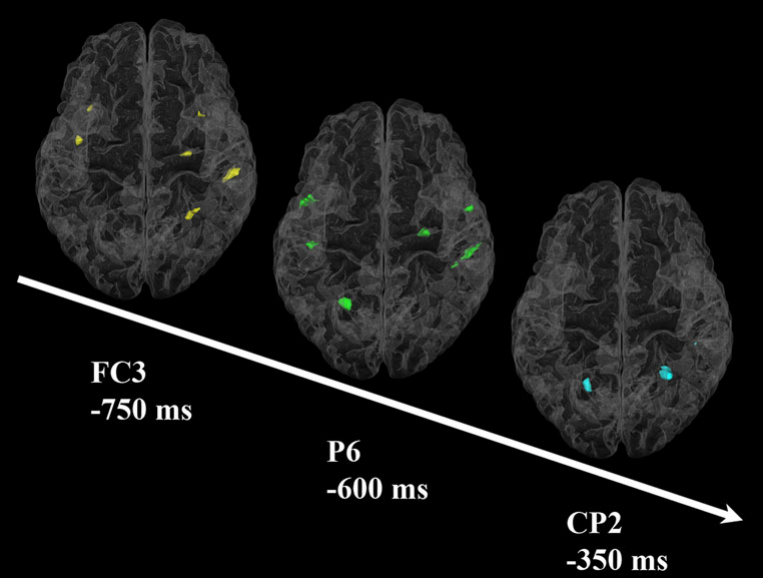
Estimated time course of neural dynamics at the FC3, P6, and CP2 electrodes. Schema of the time course of intrinsic neural dynamics represented by the three activation maps shown in Fig. 3. The FC3-group was activated 750 ms before the button was pressed, followed by activation of the P6-group at 600 ms and the CP2-group at 350 ms

Our results demonstrate that rapid neural dynamics arose before observers were aware of SPS induced by the Necker cube. EEG data simultaneously recorded during fMRI scanning and source estimates of the data showed rapid dynamics of neural substrates electrophysiologically related to SPS. In addition, our EEG–fMRI integrated analysis indicated that the oscillation-related regions were activated in accord with a sequence of 3–4 Hz EEG oscillatory modulations more in the test than in the control condition. This integrated analysis also demonstrated that a sequence of three groups of activations correlated with 3–4 Hz slow oscillatory modulations traveled from the anterior to posterior cortical regions sequentially prior to the presumable latency of SPS (Fig. 6). These findings are consistent with a previous study reporting the frontal-to-occipital synchrony or connectivity before SPS induced by the Necker cube (Shimaoka et al. 2010).

### Oscillation-related regions inside the DAN

A large-scale meta-analysis of the precise functional neuroanatomy of the DAN indicated that the following brain regions should be included in the DAN (Toro et al. 2008): the IPS, ventral IPS, inferior parietal lobule, hFEF, inferior precentral sulcus (iPrCeS), supplementary motor area (SMA), pre SMA, dorso-lateral prefrontal cortex (DLPFC), ventral occipital cortex, aIC, ventral hFEF, thalamus, and right cerebellum. In contrast, we found that the oscillation-related regions included the hFEF, IPS, vPrCeS/vPrCeS (that are included in the iPrCeS), aIC, FO, and PoCeC. Comparisons of these two sets of results indicated that almost all of the oscillation-related regions are included within the DAN, whereas only the FO and bilateral PoCeC were not. Thus, these oscillation-related regions can be regarded as parts of the DAN.

### Neural dynamics along the DAN prior to SPS

Our findings indicate that a sequence of three prominent neural activities occur prior to the Necker cube induces SPS. These neural activities showed local maxima at 750 (FC3), 600 (P6), and 350 (CP2) ms before the button was pressed, indicating that these are peak latencies in response to SPS. Since almost all of the oscillation-related regions correlating the sequence of neural activities were within the DAN (Toro et al. 2008), and since this sequence traveled sequentially from anterior to posterior regions, our results confirmed our hypothesis, that rapidly changing neural dynamics arising along the DAN from frontal to parietal regions arise prior to SPS induced by the Necker cube.

Electrophysiology has shown that right parietal activity arose 50 ms before the onset of the Necker cube stimuli (Britz et al. 2009). Similarly, our findings indicate that parietal activity appears beginning approximately 400 ms before, or approximately 50 ms before the presumable onset latency of each percept of the Necker cube. Functional neuroimaging of BOLD signal latency analysis showed that right inferior frontal activation occurred 800 ms before button pressing in response to SPS by the dynamic dot quartet, whereas the left FO, the bilateral MT+, the right inferior parietal region and the left sensorimotor cortex were not activated prior to button pressing (Sterzer and Kleinschmidt 2007), suggesting that serial processes underlie multistable perception, but not providing any details of these processes.

In contrast to these earlier studies, our findings provide novel evidence of the details of serial processes underlying SPS. Several recent studies have described neural dynamics along the DAN. For example, the monkey FEF has been shown to transfer information to the V4 during an attentional task (Gregoriou et al. 2009). Moreover, functional neuroimaging has shown that, during voluntary control of visuospatial attention, the human FEF sends causal flows to the posterior parietal cortex, including the IPS and V4 (Bressler et al. 2008; Ozaki and Ogawa 2009). Neural dynamics along the DAN may be flexibly switched in accordance with a task demand in voluntary attentional control (Ozaki and Ogawa 2009).

Despite the low time resolution of fMRI, voluntary attentional control may be illustrated by a model of neural dynamics from the anterior (the FEF and other frontal areas) to the posterior (the IPS and other parietal or occipital areas) regions along the DAN. Functional neuroimaging of resting-state networks (RSN) has also revealed intrinsic neural dynamics along the DAN, functionally dissociating the DAN from other brain regions (Fox et al. 2006; Vincent et al. 2007). Moreover, simultaneous EEG–fMRI assays of RSNs suggested that these intrinsic neural dynamics may have rapid microstates on a fine time scale (Britz et al. 2010). Our hypothesis and results are consistent with these findings and models.

Finally, one may argue that not voluntary but involuntary attentional control can contribute to SPS and that SPS should be related to some neural correlates of involuntary attentional control. However, many previous studies have revealed that cortical regions related to involuntary attentional control are clearly dissociable from those related to voluntary attentional control, because neural substrates of involuntary attentional control are mainly in the ventral part of the cerebral cortex while those of voluntary attentional control are mainly in the dorsal part (see Corbetta and Shulman 2002; Corbetta et al. 2008 for review).

**Fig. 7:**
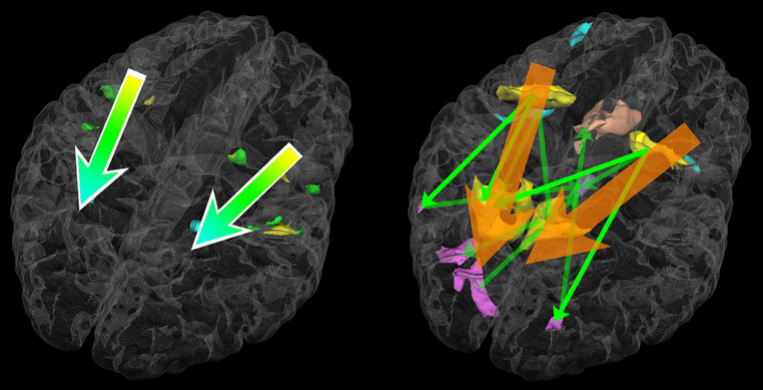
Major flows of intrinsic neural dynamics. Intrinsic neural dynamics suggested by our findings were compared with typical neural dynamics (Ozaki and Ogawa 2009). *Left* prior to pressing the button, intrinsic neural dynamics arose along the DAN from the anterior (frontal) to the posterior (parietal) regions. *Arrows* indicate the flow of processing along the DAN. *Right* neural dynamics along the DAN from the frontal to parietal regions during voluntary attentional control. *Orange arrows* indicate global flows along the DAN. This coincident flow indicates that neural dynamics are similar for voluntary attentional control and the perception of SPS. The figure on the right is reproduced from ref. (Ozaki and Ogawa 2009) with permission from © (2009) Lippincott Williams & Wilkins. (Color figure online)

In summary, our results suggest that intrinsic neural dynamics along the DAN traveling from anterior to posterior regions, similar to the dynamics observed during voluntary attentional control, can change a status of voluntary attentional control and may subsequently trigger SPS (Fig. 7).

### Interaction of the neural dynamics in the DAN revealed by the EEG–fMRI integrated analysis with representative cortical regions related to the Necker cube perception

Our EEG–fMRI integrated analysis indicated that neural dynamics before SPS travels along the DAN from the anterior to posterior cortical regions. This finding suggests that some interaction between such a traveling neural dynamics and functional structure of the DAN may occur before SPS.

As far as we have searched, there are only a few functional neuroimaging studies investigating functional localization of the neural correlates of SPS induced by the Necker cube. An earlier fMRI study by Inui and colleagues indicated that the bilateral dorsal premotor and parietal cortex would be important for the Necker cube perception (Inui et al. 2000). On the other hand, a recent study by Knapen and colleagues reported that the fronto-parietal pathway in the right hemisphere would play an important role in embodying the bistable percept itself (Knapen et al. 2011). However, these previous reports could not provide any evidence of the importance of traveling neural dynamics from the anterior to posterior cortical regions.

Perception of the Necker cube also can be related to the language function. A neuropsychological study reported that hemispatial neglect patients with higher verbal intelligence can copy the Necker cube better than patients with lower verbal intelligence (Seki et al. 2000). This fact supports a finding that the left ventral frontal cortex adjacent to the Broca’s language area was included in the FC3 group. Taken together with the finding of Knapen’s group (Knapen et al. 2011), the left ventral frontal activity reported in the current study must be more important for not the bistable perception, but SPS itself.

Thus, our results indicated that the anterior regions of the DAN and some parts of the left ventral frontal cortex supposed to be related to the language function were first activated, and that broadly-distributed regions of the DAN were activated, and that finally the bilateral posterior parietal regions within the DAN were activated. Such a neural dynamics traveling along the DAN may reflect a time course of fine-scale temporal structure of cognitive dynamics underlying SPS induced by the Necker cube.

### Origin of multistability as a fundamental framework of SPS

Unlike the attention studies cited above, our study did not include any explicit voluntary attentional control. Nonetheless, our hypothesis and results indicate that the neural dynamics along the DAN from frontal to parietal regions appeared prior to SPS during multistable perception, as well as during voluntary attentional control. These findings suggest that similar mechanisms of the DAN underlie both SPS induced by the Necker cube and voluntary attentional control.

Two possible mechanisms of the DAN may be involved in Necker cube induced SPS: flexibility and/or fluctuation of the network. In regard to flexibility, there is evidence that neural oscillations across various frequency bands mediate self-organization in the human brain. For example, neural oscillations across broad frequency bands recorded by magnetoencelphalography (MEG) make small-world networks emerge dynamically and flexibly in response to task demands (Bassett et al. 2006). Indeed, theoretical studies have proposed that oscillatory phenomena involve a similar mechanism of self-organization of neural oscillators through synchronization (Shimizu and Yamaguchi 1987; Yamaguchi and Shimizu 1994). This hypothesis suggests that the FC3-, P6- and CP2-groups correlating with various kinds of oscillations may be embodied by a process of self-organization of neural oscillators and/or synchronization.

In contrast, theoretical studies have suggested various approaches to the fluctuation of networks. For example, neuronal fluctuations may be a source of stochastic behavior, i.e. multistability (Deco and Romo 2008). These fluctuations may lead to stochastic transition of a status of systems, i.e. stochastic resonance (Kitajo et al. 2003; Ward et al. 2006). Our findings suggest that the three activation groups appearing prior to SPS are mediated by neural oscillations, and that, as a network, they may easily transit from one to another by means such as stochastic resonance, together with some intrinsic noises in the human brain. Moreover, our findings suggest that such a transition through the three activation groups may result in a sequence of neural dynamics along the DAN from frontal to parietal regions.

There is considerable evidence about intrinsic and noise-induced fluctuations in the human brain (Fox et al. 2005; Fox and Raichle 2007; He and Raichle 2009), especially in the DAN (Fox et al. 2006; Vincent et al. 2007). This evidence indicates that intrinsic and noise-induced fluctuations in the DAN may give rise to sub-networks and embody neural dynamics along the DAN as a global flow. This would lead to fluctuations in voluntary attentional control, thus triggering SPS, especially by the Necker cube.

In contrast to binocular rivalry, ambiguous perception may be modulated by attentional or intentional control, although both are multistable (Meng and Tong 2004). Our findings may explain the difference between ambiguous perception by single figures and binocular rivalry. That is, ambiguous perception is mediated by self-organization and/or stochastic resonance of neural oscillations involved in attentional control that travel along the DAN, whereas binocular rivalry, although a similar stochastic process, arises mainly in the visual cortex and partly in a fronto-parietal circuit but not in the DAN (Sterzer and Rees 2008).

In conclusion, simultaneous EEG and fMRI evaluations of participants performing a multistable perception task with the Necker cube identified intrinsic neural dynamics driven by 3–4 Hz slow oscillations along the DAN from frontal to parietal regions prior to SPS induced by the Necker cube, thus confirming our working hypothesis. Our results indicate that such intrinsic neural dynamics may trigger SPS, and that they can be driven by self-organization and/or stochastic resonance of slow neural oscillations. These findings provide a novel explanation about why ambiguous perceptions can be modulated by voluntary attentional control whereas binocular rivalry cannot: the former is driven by a stochastic process in the DAN, whereas the latter is driven by a similar stochastic process but not within the DAN.
